# Resonant nano-dimer metasurface for ultra-thin a-Si:H solar cells

**DOI:** 10.1038/s41598-021-86738-6

**Published:** 2021-03-30

**Authors:** Mahmoud H. Elshorbagy, Pablo A. Sánchez, Alexander Cuadrado, Javier Alda, Óscar Esteban

**Affiliations:** 1 Photonics Engineering Group, University of Alcalá, Alcalá de Henares, 28801 Madrid, Spain; 2 Physics Department, Faculty of Science, Minia University, El Minya, 61519 Egypt; 3 Escuela de Ciencias Experimentales y Tecnología, University Rey Juan Carlos, Móstoles, 28933 Madrid Spain; 4 Applied Optics Complutense Group, University Complutense of Madrid, Arcos de Jalón, 118, 28037 Madrid, Spain

**Keywords:** Energy science and technology, Optics and photonics

## Abstract

Low-cost hydrogenated amorphous silicon solar cells (a-Si:H) can perform better and be more competitive by including nanostructures. An optimized nano-dimer structure embedded in close contact with the back electrode of an aSi:H ultra-thin solar cells can enhance the deliverable short-circuit current up to 27.5 %. This enhancement is the result of an increase in the absorption at the active layer, that is the product of an efficient scattering from the nanostructure. From our calculations, the nano-dimer structure must be made out of a high-index of refraction material, like GaP. The evaluation of the scattering and absorption cross section of the structure supports the calculated enhancement in short-circuit current, that is always accompanied by a decrease in the total reflectance of the cell, which is reduced by about 50 %.

## Introduction

The photovoltaic industry aims to develop novel technologies that simultaneously increase efficiency and reduce fabrication costs. High-precision thin-film coating technologies at large scale^1–4^ promote a new generation of photovoltaic cells that requires intensive analysis in modeling, fabrication, and testing to optimize its performance^5,6^. This generation of thin-film solar cells is the low-cost alternative to the thick wafer-based high-cost devices^7,8^. Thick wafer-based solar cells require high purity, single-crystalline materials to produce efficient devices^9,10^. Whereas, thin-film solar cells can be fabricated using less raw materials with lower purity and crystallinity requirements without reducing their performance^11,12^. Both inorganic and organic materials have been proposed for thin-film solar cells^11,12^; but if we consider stability and lifetime, inorganic semiconductors perform better for solar cells applications^13,14^. Among inorganic materials, hydrogenated amorphous silicon (a-Si:H) is a reliable candidate for large scale fabrication because it is an abundant and non-toxic material^15,16^. However, a-Si:H still has some challenges to overcome. For instance, the low mobility and short lifetime of their charge carriers reduce the probability of their collection at the device electrodes^17^. A possible solution is to thinner the a-Si:H active layer up to a maximum thickness of $\approx$ 300 nm^18–20^. Another limitation of a-Si:H is the photo-induced performance degradation—also known as the Staebler–Wronski effect (SWE)^21^—that heals with thermal annealing^22–26^. Once the absorption at the active layer is optimized, if the solar cell design increases the absorption at the auxiliary layers of the cell, the temperature increases and helps to mitigate the SWE defects^26,27^.

Different strategies have been adopted to reduce the reflectance^28,29^, and increase the absorption efficiency^30,31^, and the short-circuit current density $J_{\mathrm{SC}}$, delivered by thin-film solar cells^16,32–34^. Our approach increases the optical path of light within the active layer to generate more charge carriers. To do that without disturbing the layer’s arrangement of the cell, we study the role of the back contact of the ultra-thin solar cell. In thin-film solar cells, it is located very close to the cell’s surface and a large amount of light reaches it and is reflected back specularly, allowing light to escape without generating charge carries. To reduce the high reflectance of ultra-thin solar cell, many back texture and nanostructures have been applied to diffusely reflect the light that reaches the back contact. The reported strategies vary from the addition of silver nanoparticles^35^, plasmonic gratings^36^, textured substrates^37^, distributed Bragg reflectors^38^, and high-aspect ratio grating to serve as scaffold for conformal growing of ultra-thin solar cells^39^. Similar additions can be used to produce in-situ annealing of the device^26,27^ that helps to repair SWE defects. These solutions can be classified in two: highly ordered patterns, and random textures. Meanwhile the randomly organized nanostructures are easier to fabricate, they are less reproducible and may induce scattering of the charge carriers^40,41^. In solar cells, comparative analysis of both solutions tend to select periodic nanostructures as better suited for improved devices^42^, although a controlled increase of the disorder also benefits their performance^43^. Our design can be labeled as highly ordered and should benefits from the effects presented in previous publications^42^. As a summary, the improvement of a thin a-Si:H solar cell presented in this paper is based on (i) enhanced absorption at the active layer by incorporating a nanophotonic structure that also reduces its total reflectance; (ii) increase of the probability of charge carrier collection, and decrease in the number of defect states arising from SWE with a thinner active layer; and (iii) improved absorption at the cell’s auxiliary layers of the cell that will mainly converted to heat mitigating the SWE defects. These characteristics should be extended over the broadband spectral range where the solar cell operates.

To take advantage of an optimized structure, our approach departs from an ultra-thin aSi:H solar cell. Then, the back contact is modified to increase the amount of light available at the active layer, without compromising the isolation of the metal from the active layer because the nanostructure is embedded with the transparent conductive oxide layer. To do that efficiently, we first select the material of the proposed nano-dimmers. This material should have a higher index of refraction and a negligible absorption. As far as the periodicity of the nanostructures modifies its optical response and its performance as a photovoltaic device, our study also considers the geometrical parameters of the nanostructure. The values of these parameters have been selected to assure the fabrication of the device. Also, the performance of the cell has been analyzed under realistic conditions of illumination in terms of the angle of incidence and polarization state.

## Materials and methods

The practical realization of the proposed nanostructure is a nano-dimer metasurface that is embedded in the transparent buffer layer at the back side of the cell, between the thin n-type layer and the back metallic electrode. This design scatters light towards the active layer of the cell, and additionally resonates at specific wavelengths. The standard material arrangement of the planar device is preserved, and the proposed design does not affect the charge carrier transport within the device. After presenting and discussing the design, we describe the optimization procedure that maximizes the photo-generated current, $J_{\mathrm{SC}}$. In this optimization, we included practical limitations in the feasibility of the fabrication using available technologies and processes, such as e-beam lithography and nanoimprint techniques.

Our design is an adaptation of the layer structure of a thin a-Si:H solar cell^44–46^, and is listed in Table 1 from bottom to top for a ultra-thin solar cell.

**Table 1:** Layer structure of ultra thin a-Si:H solar cell (from bottom to top).

Layer (shape) ${}^{\mathrm{Reference}}$	Thickness (nm)
Silver, Ag (thin-film)^47^	$t_{\mathrm{Ag}}= 200$
Indium zinc oxide, AZO (thin-film)^48^	$t_{\mathrm{AZO}}=100$
n-type aSi:H, n-aSi:H (thin-film)^49^	$t_{\mathrm{n-aSi:H}}=22$
i-type aSi:H, i-aSi:H (thin-film)^49^	$t_{\mathrm{i-aSi:H}}=150$
p-type aSi:H, p-aSi:H (thin-film)^49^	$t_{\mathrm{p-aSi:H}}=17$
Indium tin oxide, ITO (thin-film)^50^	$t_{\mathrm{ITO}}=70$
Glass, $\hbox {SiO}_{2}$ (substrate)^51^	$t=\infty$

**Figure 1:**
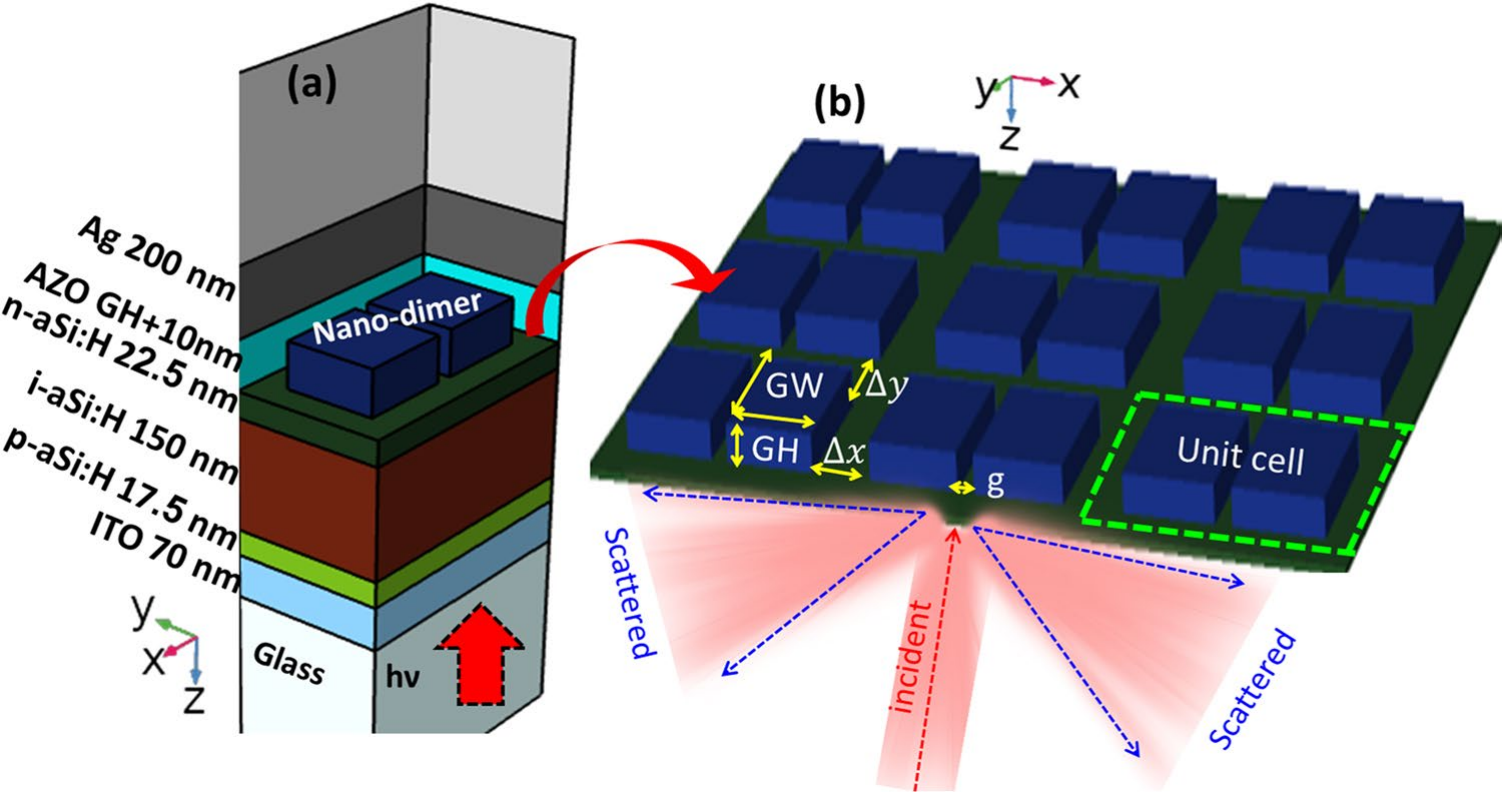
(**a**) Geometrical arrangement of the layers of an ultra-thin-film aSi:H solar cell. (**b**) Geometrical parametrization of the nano-dimer metasurface. Both 3D plots in a,b were generated using the COMSOL software.

For a standard design of a thin a-Si:H solar cell, the back contact acts as a mirror that reflects the incident light specularly. This bouncing trajectory travels a short distance through the active layer of the cell that, in our case, is only 150 nm thick. This reduced thickness is an advantage when collecting the photo-generated charge carriers, but it is not optimum in terms of the optical absorption of the active layer. To increase such optical path, our design includes dielectric nano-dimers embedded in the AZO layer and located in close vicinity to the metallic layer. This addition modifies the angular distribution of the light reflected from this back contact. By doing that, light scatters towards the active layer to photo-generate more charge carriers. The arrangement of this design is generated using COMSOL in Fig. 1a and a 3D view of the structure is also shown in Fig. 1b. Actually, between the AZO layer and the active layer, we find a 22 nm thick n-type aSi:H that has a negligible optical effect.

As far as the nanostructure is embedded in the AZO layer, its scattering efficiency is larger as the contrast of the index of refraction increases. This analysis has been made and it is explained in "Optimization" section. Here, we can advance that GaP is an appropriate candidate to fabricate the nano-dimers. The geometry of the unit cell of the structure is made of two cuboids with square section, with side GW, and separated by a gap distance *g*, being GH its height. The cuboids are aligned along the *X* direction. The distance between adjacent dimers is $\Delta x= \Delta y$ along *X* and *Y* directions, respectively. All together, the dimers are arranged with the following spatial periods along both directions (see Fig. 1b):

1 $$\begin{aligned} P_x&= {} 2 \mathrm{GW} + \Delta x + g, \end{aligned}$$

2 $$\begin{aligned} P_y&= {} \mathrm{GW} + \Delta y. \end{aligned}$$

These equations show how the periodicity of the structure is linked to the geometrical parameters that we will use in our optimization procedure. Therefore, when changing variables $\text{ GW }$ and *g*, the effect of this variation on the electromagnetic response is taken into account.

Using a computational electromagnetism tool, we have evaluated the optical performance of the device. COMSOL Multiphysics, based on the Finite Element Method (FEM), is used to compute the 3D distribution of the optical fields into each layer of the cell by solving Maxwell equations. The model uses an optical source launched from a port placed at the bottom of the device (glass side). The source generates plane waves which spectral power distribution resembles the standard solar irradiance $\Phi _{\mathrm{AM1.5}} (\lambda )$^52^. The orientation and polarization of these plane waves are selected to cover the operation conditions of the cell. The computation is made in the unit cell presented in Fig. 1a. Periodic boundary conditions are implemented at the lateral sides of the unit cell to account for the whole device. Another listening port is placed on top of the whole structure, with the proper orientation according to the incident wave conditions. Both input and output ports serve to calculate the total reflection and total transmission of the system. They are located farther away from the structure to avoid unwanted effects. A built-in function calculates the spectral absorbed power for each layer. Perfectly matched layers (PML), added to the top and bottom of the structure, prevent interference effects between the incoming and reflected radiations. The results for the unpolarized sun radiation is obtained by averaging the two polarization components. This is a critical point because, even in normal incidence conditions, the nano-dimer structure is not isotropic and the results are not equivalent for TE and TM field orientations. The variable of interest is the short-circuit current, $J_{\mathrm{SC}}$, delivered by the device and calculated as^53^:

3 $$\begin{aligned} J_{\mathrm{SC}} = \int _{\lambda _{\mathrm{min}}}^{\lambda _{\mathrm{max}}} \left( \frac{q \lambda }{hc} \right) A(\lambda ) \Phi _{\mathrm{AM1.5}} (\lambda ) d\lambda , \end{aligned}$$

where $A(\lambda )$ is the spectral absorption within the active layer, *q* is the electron charge, *c* is the speed of light in vacuum, and *h* is the Planck’s constant. The integration is carried out over the range $\lambda \in [300, 800]$ nm, where i-aS:H absorbs. This equation assumes that every absorbed photon generates a couple of charge carriers contributing to the delivered current. The evaluation of this spectral absorption, $A(\lambda )$, is based on the following equation^53,54^:

4 $$\begin{aligned} P(\lambda ) = \frac{\pi c \varepsilon ^{\prime \prime }}{\lambda } \vert E(\lambda )\vert ^2 , \end{aligned}$$

where $\varepsilon ^{\prime \prime }$ is the imaginary part of the dielectric permittivity of the material, and $\vert E(\lambda )\vert ^2$ is the square of the electric field intensity inside each material. As far as $P(\lambda )$ is evaluated at each location of the structure, it is possible to obtain the total absorbed power contributing to the short-circuit current just by integrating $P(\lambda )$ within the volume of interest, in this case, the active layer. Dividing the total absorbed power in a specific layer by the input power, we get the spectral absorption in this layer, A($\lambda$). Also, this method can be used to evaluate the contribution of the sun irradiance to the thermal dissipation within the cell, that could help to mitigate the SWE. This optical model has been validated against numerical and experimental results in several previous contributions^16,27,39,55,56^.

### Optimization

In this section we describe the optimization process for the structure. Here, we also check how the final optimum design complies with the expected results for the value of the index of refraction of the dimer structure. We have also analyzed the role of the geometrical parameters of the nano-dimer unit cell. From a computational point of view, we have used Matlab in combination with COMSOL to drive the optimization process. Then, we have taken advantage of both tools to calculate the short circuit current, and the reflectance maps.

The first step in the optimization is to find a material achieving a high refractive index contrast with AZO ($n_{\mathrm{AZO}} \approx 1.9$ at $\lambda = 600$ nm^48^) that promotes an efficient scattering. Afterwards, the geometry of the nanostructure tunes this scattering at the wavelengths of interest. In practice, this means two successive optimization processes: one for the material of the dimers, and a second one for the dimer geometry using the previously selected material.

There exist several suitable materials to build the dimers. Although dielectric, the foreseeable materials have a refractive index that is complex, ${\tilde{n}}=n+i\kappa$, with an almost negligible imaginary part, and a smooth spectral variation of the real part in the visible range. This is why, in this step, we have neglected the imaginary part of the refractive index. In order to consider almost any possible material, the real part of the index of refraction is scanned from 1.3 till 4.0. The optimization process maximizes the short circuit current, $J_{\mathrm{SC}}$. At the same time, we have analyzed the total reflectivity of the cell *R* in the wavelength range $\lambda$ $\in [300, 800]$ nm. To provide reasonable geometry parameters, we have made this calculation by fixing $\Delta x= \Delta y = 200$ nm, $g=20$ nm, and $\mathrm{GH}=100$ nm, meanwhile $\mathrm{GW}$ can be changed in the optimization to already include a geometrical variable in the calculation. All these geometrical parameters are linked through Eqs. (1) and (2).

**Figure 2:**
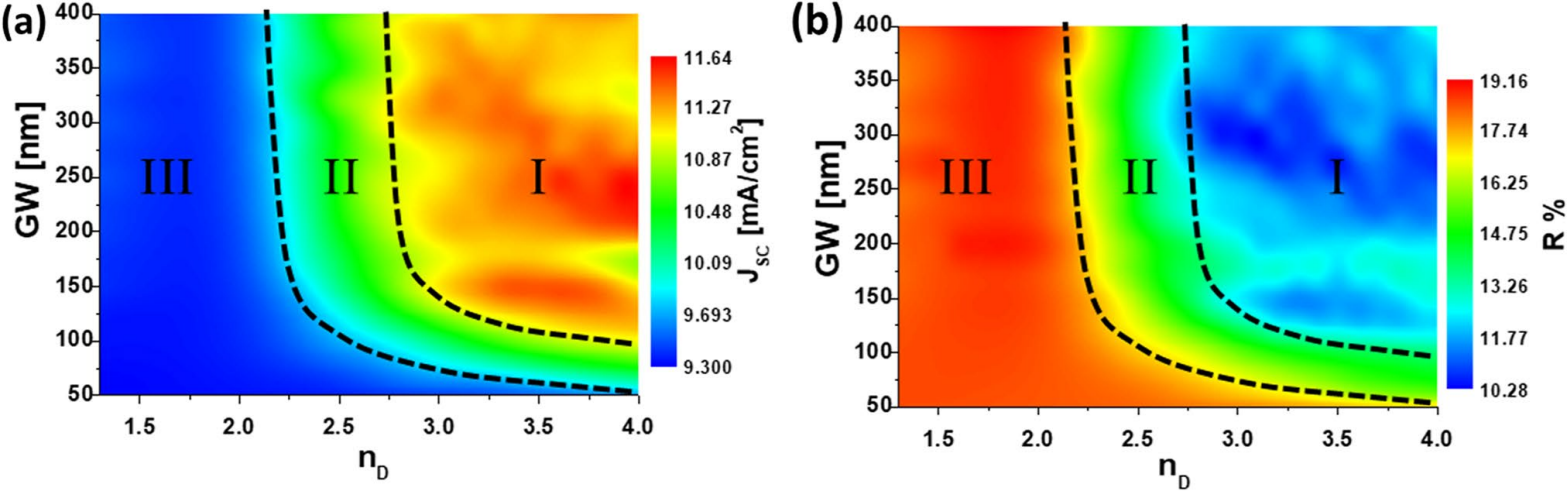
Maps of the short-circuit current (**a**) and total reflectance (**b**) in terms of the index of refraction of the dimer’s material and the lateral size, GW, of the cuboid element. The reflectance of the whole device, *R*, is calculated in the range $\lambda \in [300, 800]$ nm. The dashed curves define three regions in performance.

Figure 2 represents the maps of $J_{\mathrm{SC}}$ and *R* as a function of the real part of the index of refraction of the dimer, $n_{\mathrm{D}}$, and the lateral size of the elements of the dimer, $\mathrm{GW}$. We can identify three regions of high, moderate, and low enhancement in the $J_{\mathrm{SC}}$. It is important to note that a larger short-circuit current should be associated with a low reflectance of the cell. Also, we recall that the unmodified solar cell provides a reference value of $J_{\mathrm{SC,ref}}=9.14$ mA/$\hbox {cm}^2$, and $R_{\mathrm{ref}}=18$%. In Fig. 2a, region I shows a high enhancement in the $J_{\mathrm{SC}}$ up to 26.5 % compared with the planar solar cell without the dimer structure. This region corresponds with high refractive index materials, $n_{\mathrm{D}} > 3$. Region II comprises $2.2< n_{\mathrm{D}} < 3$, that shows a lower contrast in the index of refraction with respect to the AZO layer, and consequently, it produces a moderate enhancement of about 15 % in $J_{\mathrm{SC}}$. In region III ($n_{\mathrm{D}} < 2.2$) we can find materials with refractive index comparable or lower than AZO layer. In this later case, no $J_{\mathrm{SC}}$ enhancement is found. The same results are obtained when analyzing the map in Fig. 2b where *R* is represented. As expected, if the reflectance of the cell decreases, light is better absorbed and more charge carriers are contributing to $J_{\mathrm{SC}}$. Actually, reflectance varies from around 20 % in the case of the planar device, to about 10 % in region I (this is a 50 % drop).

Many materials have a refractive index within region I, such as gallium phosphide (GaP), gallium arsenide (GaAs), silicon (Si), etc.^57^. The actual selection should consider those materials with low absorption in the long wavelength portion of the spectrum ($\lambda \in [500, 700]$ nm), because shorter wavelengths will be mainly absorbed in the front layers (ITO, p-aSi:H, i-aSi:H, and n-aSi:H). Therefore, only longer wavelengths reach the back side of the cell. GaP fulfills this characteristic, being its refractive index around 3.5 in the visible region^57^ with low absorption losses at wavelengths longer than 500 nm.

Once the material has been selected, we continue the analysis using the actual complex refractive index of GaP^58^. The next optimization step is related with the dimer geometry. In this case, $\mathrm{GW}$ and $\Delta x$ are the free parameters that can vary to obtain the two dimensional maps of $J_{\mathrm{SC}}$ and *R*. The goal of this step is to provide a feasible and flexible combination of geometrical parameters that could be used to fabricate optimum devices.

**Figure 3:**
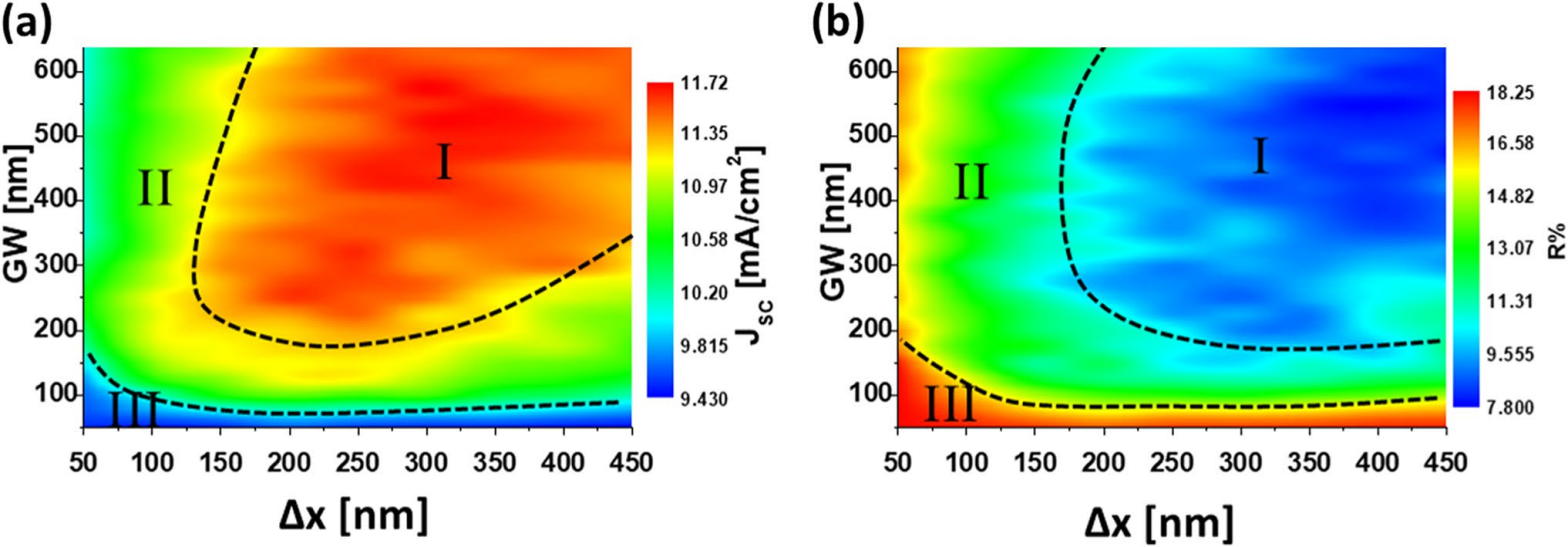
Maps of the short circuit current, $J_{\mathrm{SC}}$ (**a**), and the reflectance, *R* (**b**), within the spectral range $\lambda \in (300, 800)$ nm, in terms of $\Delta x$ and $\mathrm{GW}$ geometrical parameters. The material of the metasurface elements is GaP.

As we can see in Fig. 3a the map is divided again into three regions of different enhancement values of $J_{\mathrm{SC}}$. In this map, region I, corresponding to a high enhancement of $J_{\mathrm{SC}}$ (up to 27.5 %), is very wide and covers almost all the evaluated dimer widths ($\mathrm{GW} > 200$ nm) and separation distances ($\Delta x= \Delta y > 150$ nm). Region II provides a moderate enhancement in $J_{\mathrm{SC}}$ up to 15 %, while the values that leads to almost no enhancement are located within a very small region III. As it happened in the first step of the optimization, the reflectance map (see Fig. 3b) agrees well with the $J_{\mathrm{SC}}$ enhancement map to confirm again the power balance behind this behavior: as $J_{\mathrm{SC}}$ increases (absorption increases), *R* decreases. From these results, we can select an optimum geometry from the wide combination of the $\Delta x$ and $\mathrm{GW}$ geometrical parameters in region I that leads to enhanced $J_{\mathrm{SC}}$ values. Therefore, we select a suitable combination of $\Delta x = \Delta y = 300$ nm, and $\mathrm{GW}= 575$ nm to build GaP nano-dimers.

**Figure 4:**
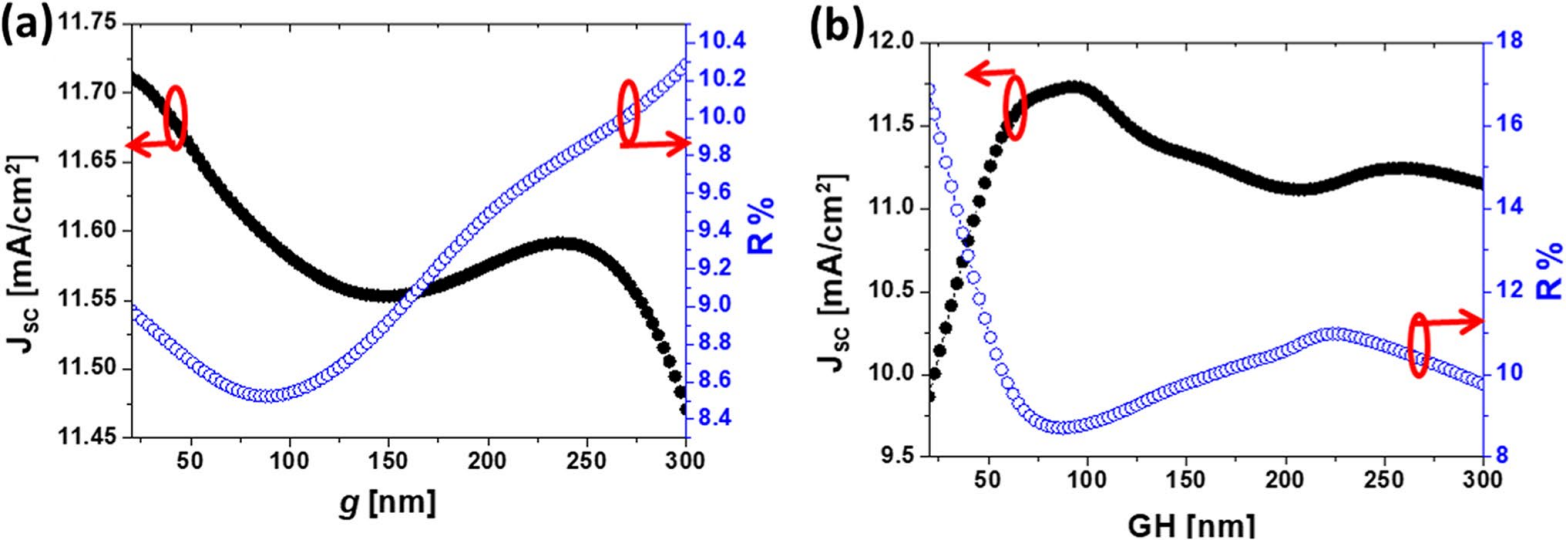
Variation of the short circuit current, $J_{\mathrm{SC}}$, and reflectivity, *R*, as a function of the gap distance, *g*, (**a**), and as a function of the height of the dimer elements, $\mathrm{GH}$, (**b**). The material of the dimer structure is GaP. The reflectance is calculated for the spectral range between 300 and 800 nm.

After these two optimization steps, a suitable combination of $n_{\mathrm{D}}$, $\Delta x$, $\Delta y$, and $\mathrm{GW}$ improves significantly the solar cell performance. However, there are still two remaining parameters that have been kept constant along these calculations: the dimer separation *g*, and the cuboid’s height $\mathrm{GH}$. The lower limit, $g=20$ nm, is selected after considering manufacture limitations. In Fig. 4a, we can see how $J_{\mathrm{SC}}$ decreases rapidly for gap distances from 20 nm to 100 nm, begin almost constant in the range $100< g < 250$ nm, and decreasing again very sharply for gap distances close to 300 nm. This behavior can be explained by the decoupling between the dimers elements as the gap widens. However, enlarging the gap from 20 to 250 nm only decreases the $J_{\mathrm{SC}}$ from 11.7 to 11.55 mA/$\hbox {cm}^{2}$ which is still a 25.5 % enhancement compared with the planar device. Having $g=300$ nm, equals the gap width to the optimized $\Delta x$ and $\Delta y$ values, that still represents a 24 % $J_{\mathrm{SC}}$ enhancement. In this last case, the dimer becomes a single element. Consequently, the dependence of $J_{\mathrm{SC}}$ with respect to *g* doesn’t show a large variation. Actually, changing *g* from 20 to 300 nm leads to 4 % variation in $J_{\mathrm{SC}}$. However, if we pay attention to the reflectance variation in terms of *g*, we find that this parameter varies more than 15 %. This change in reflectivity, in combination with an almost constant value of the short-circuit current, means that the optical irradiance is absorbed by the auxiliary layers in the cell, contributing to an increase in temperature that helps to mitigate SWE defects.

Moreover, as we see in Fig. 4b, dimer heights below 50 nm lead to a large decrease in $J_{\mathrm{SC}}$. From this calculation, the optimum heights that provide enhancement more than 25 % are from 50 to 110 nm, and $J_{\mathrm{SC}}$ starts to decrease again for higher values of $\mathrm{GH}$. Thus, the final optimized geometrical parameters are presented in Table 2 for nano-dimers made of GaP. A way to fabricate this structure starts with a multilayer stack of a-Si:H cell, which can be grown on a glass substrate coated with ITO using plasma-enhanced chemical vapor deposition. The pattern of the optimized nanodimer is applied onto the previous multilayer by using nanoimprint or electron beam lithography techniques. The next step is to evaporate a 100 nm GaP layer on this pattern, followed by a lift-off process. The AZO layer can be spin-coated on the nanostructured bottom surface of the cell, overfilling the nanodimer to create a flat surface where the back metal electrode is deposited to. In fact, the optimization procedure has been constrained by the feasibility of the fabrication using standard nanofabrication tools^59–61^.

**Table 2:** Values of the geometrical parameters of the nano-dimer structure, made of GaP.

Geometrical parameter	Value (nm)
GW	575
GH	100
$\Delta x = \Delta y$	300
*g*	50
$P_x$	1500
$P_y$	875

## Results and discussion

**Figure 5:**
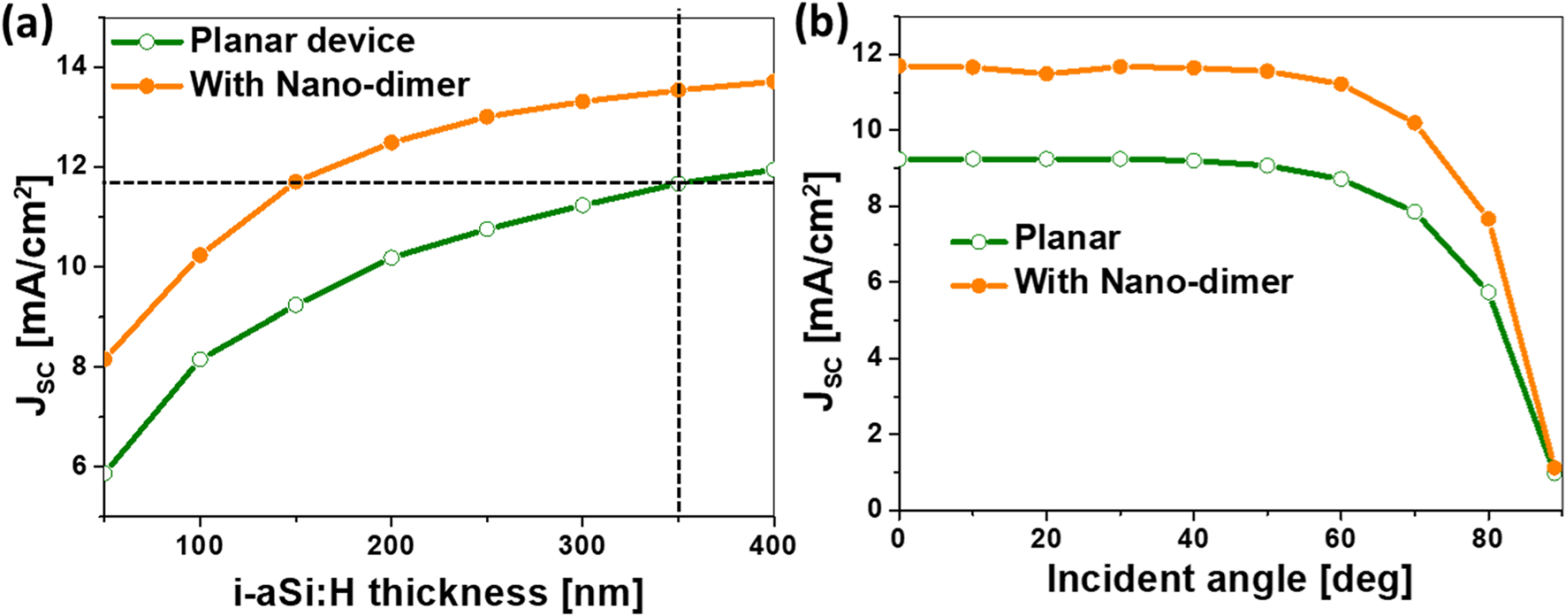
(**a**) Variation of the short-circuit current, $J_{\mathrm{SC}}$, for the optimum design (filled circles) and the unmodified planar cell (open circles) as a function of the thickness of the active layer, $t_{\mathrm{i-aSi:H}}$. (**b**) Short-circuit current as a function of the angle of incidence for the optimum design of the cell plus the nano-dimer (filled circles), and the planar solar cell (open circles).

Once the metasurface has been analyzed and optimized, we present in this section the results of the optical response of the proposed device, and how the scattering of the nano-dimer structure supports the performance improvement of the solar cell. First, we evaluate $J_{\mathrm{SC}}$ in terms of the thickness of the active layer. This analysis has been done and compared with the result obtained for a planar cell (without the nano-dimer). Figure 5a shows that, for any value of the thickness of the active layer, the dimer structure produces a higher $J_{\mathrm{SC}}$. The thickness considered in this paper, $t_{\mathrm{i-aSi:H}}=150$ nm, in combination with the optimum nano-dimer structure generates the same short-circuit current than a standard planar device with a thickness $t_{\mathrm{i-aSi:H}}=350$ nm. The higher absorption efficiency, that translate in higher short-circuit current, also leads to a larger collection probability of the photogenerated charge carriers even though the active layer is thinner. This supports the advantages of nanophotonic designs versus an increase of the active layer thickness. But even in the case of thicker active layers, the addition of the proposed nanostructure boosts $J_{\mathrm{SC}}$ around 25 %.

In solar application, we also need to know the dependence of the cell performance as a function of the angle of incidence on the cell (this is important for low-cost solar cells, where active tracking is not considered). This analysis compares the optimized device with respect to the unmodified solar cell. In Fig. 5b, we can see how the enhancement in $J_{\mathrm{SC}}$ is maintained for every angle of incidence up to 80$^{\circ }$. This happens because the nanostructure scatters light at different angle of incidence from the back electrode, which is not the case for a planar design.

**Figure 6:**
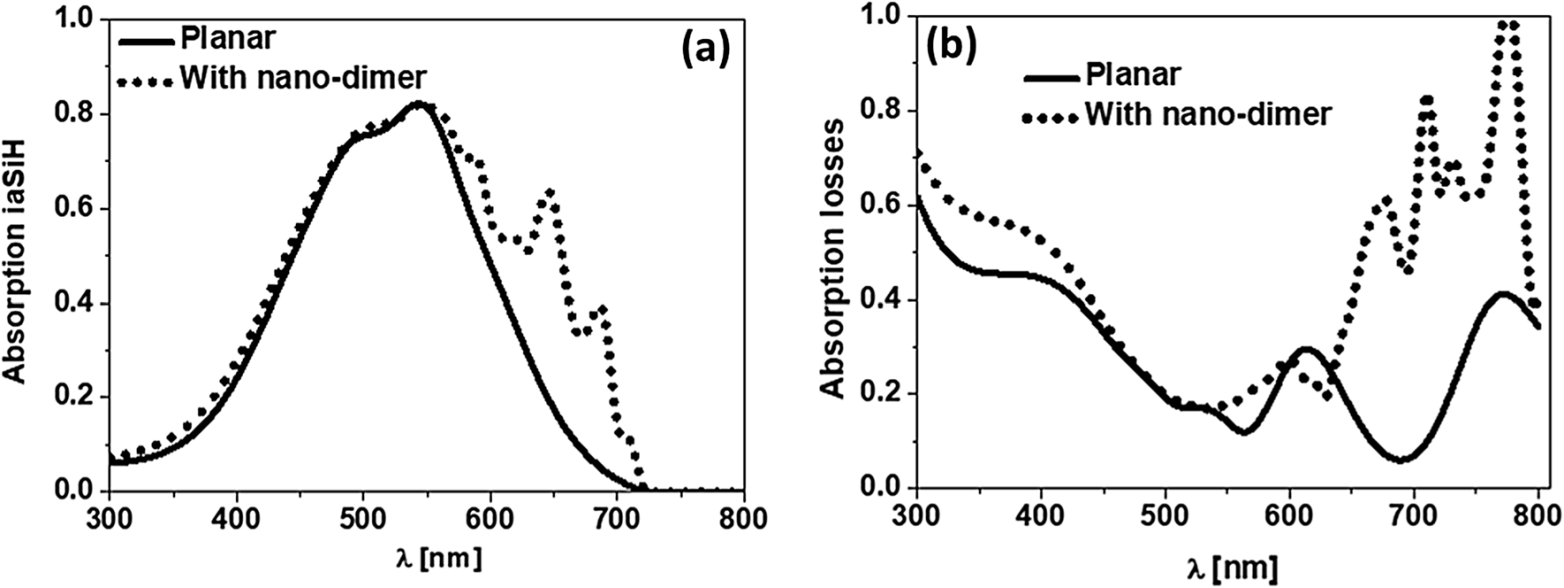
(**a**) Spectral absorption at the active i-aSi:H layer for the planar cell (solid black line), and the cell with dimers (dotted black line). (**b**) Spectral absorption at the auxiliary and buffer layers of the cell (absorption losses), for the planar cell (black solid line), and cell with dimers (black dotted line).

The optical response of the optimized design can be analyzed spectrally to understand better the advantages of including the nano-dimer structure. In Fig. 6a we can see the large enhancement in absorption within the active layer at longer wavelengths, comparing the optimum design with respect to the planar design. The enhancement is larger at the absorption edge of the i-aSi:H layer (GaP becomes almost completely dielectric above $\lambda =500$ nm), where the effect of the back contact becomes relevant. The reflectance of the cell with metasurfaces (see Fig. 3b) is lower than the reflectance of the planar device. Part of the trapped light is translated to an increase in absorption at the active layer, and also as an increase of the absorption at other layers (see Fig. 6b).

**Figure 7:**
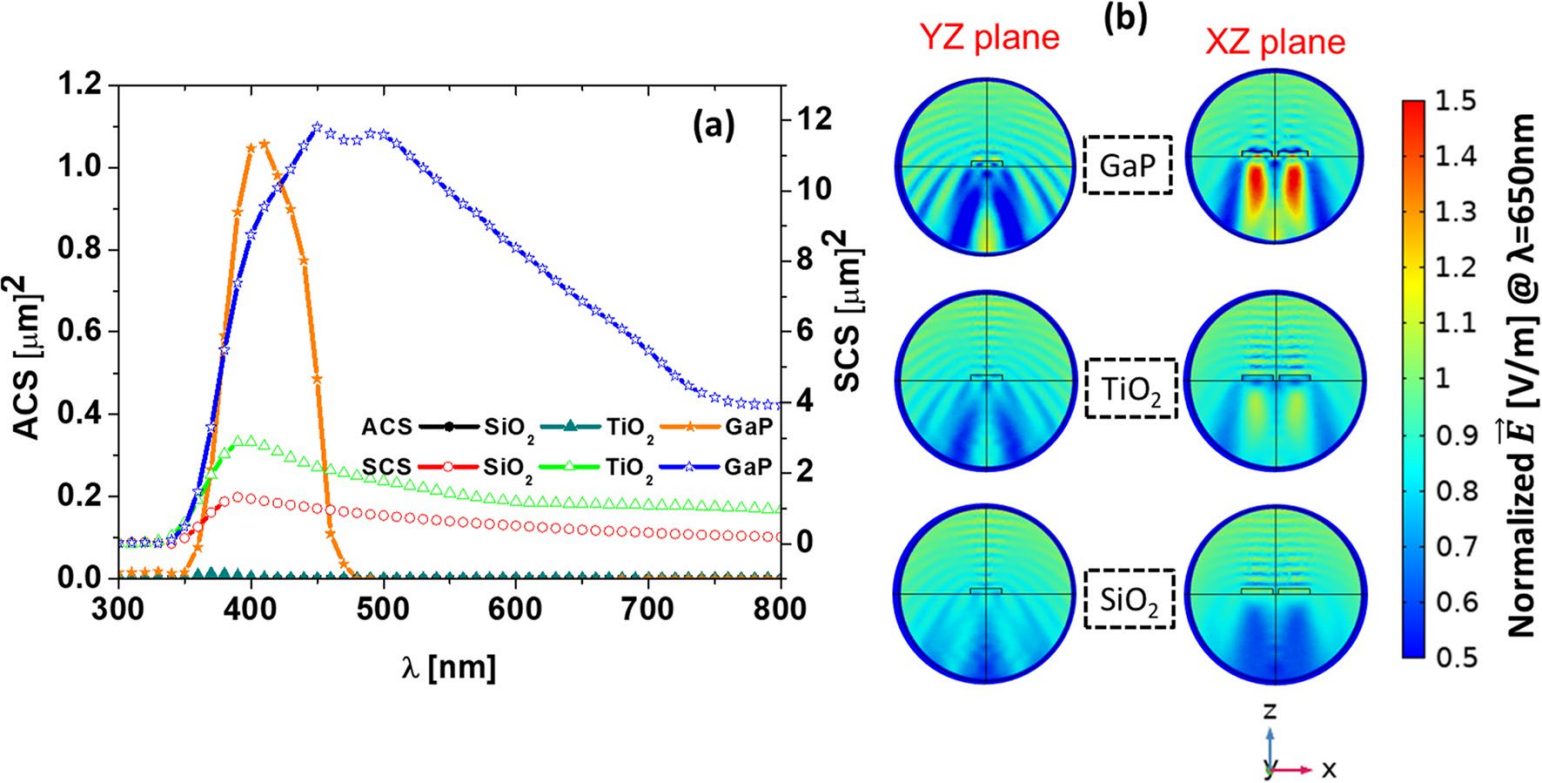
(**a**) Scattering cross section of the optimum nano-dimer (see the geometrical parameters in table 2) dimer nanoaprticle (dimer element width GW=575nm, and gap 50 nm) made from $\hbox {SiO}_{2}$ (red solid line with open circles), $\hbox {TiO}_{2}$ (green solid line with open up triangles), and GaP ( blue solid line with open stars). The corresponding absorption cross section are shown as $\hbox {SiO}_{2}$ (black solid line with closed circles), $\hbox {TiO}_{2}$ (dark green solid line with closed up triangles), and GaP ( orange solid line with closed stars). (**b**) Spatial distribution of the normalized scattered electric field of the dimer for the three materials (GaP, $\hbox {TiO}_2$, and $\hbox {SiO}_2$), for the two orthogonal planes of interest (*XZ* and *YZ*) at $\lambda =650$ nm.

To understand better the physical mechanism behind the enhancement in absorption caused by the addition of the nano-dimer structure, we calculate the scattering and absorption cross sections of the optimized dimer nanoparticle embedded in AZO. The results are evaluated for three materials: $\hbox {SiO}_{2}$ ($n_{\mathrm{SiO}_2}=1.46$), $\hbox {TiO}_{{2}}$ ($n_{\mathrm{TiO}_2}=2.5$) , and GaP ($n_{\mathrm{GaP}}=3.5$), that are located in regions I, II, and III of Fig. 2, respectively. Figure 7a plots both the absorption cross section (ACS), left axis, and the scattering cross section (SCS), right axis. As expected, the high refractive index contrast offered by GaP produces a high scattering efficiency compared with the other two materials ($\hbox {SiO}_{2}$, and $\hbox {TiO}_{2}$). In Fig. 7b, we show the spatial distribution of the scattered field for each plane (*YZ* at the left and *XZ* at the right) for GaP, $\hbox {TiO}_{2}$, and $\hbox {SiO}_{2}$ at $\lambda =650$ nm. From these near field maps we can see that GaP scatters more in every direction.

## Conclusions

We have designed and optimized a metasurface for efficient scattering in ultra-thin solar cells. It is made of nano-dimers fabricated with a high refractive index material. We have taken advantage of the structure of the back side of a standard a-Si:H ultra-thin solar cell that consists of a bi-layer made of Ag/AZO. This back electrode produces a high reflectance at longer wavelengths, and a low absorption efficiency of the device. To overcome this issue, we introduce an efficient light scatterer—a nano-dimer embedded in the AZO layer—to diffuse light and confine it in the active layer of the cell. We selected the material carefully to meet the requirements of high contrast in the refractive index contrast necessary to increase scattering. wWe found GaP suitable for our design after tuning the geometrical parameters of the nano-dimer structure. The short-circuit current is enhanced by 27.5 % mainly due to the 50 % reduction in reflectance, indicating that part of the trapped light is shared by other layers of the cell which will mainly converted to heat by dissipation. These absorption losses in the form of heat will produce in-situ annealing of the device and help to partially mitigate the defects produced by the Staebler-Wronsky effect in a-Si:H thin-film solar cells.

Our analysis shows that the structure incorporating the nano-dimer is more efficient than the planar case under all studied conditions: spectral response, thickness analysis, and angle of incidence analysis. Moreover, we verified that the scattering of the nano-dimer structure is the physical mechanism supporting the previously obtained results in $J_{\mathrm{SC}}$. The design presented in this contribution helps to better understand the role of the back contact of ultra-thin solar cells, and how a customized nanostructure boosts its performance significantly.
